# Severe hypophosphatemia induced by excessive production of FGF23 in acute hepatitis: from bedside to bench

**DOI:** 10.1093/ckj/sfae307

**Published:** 2024-10-09

**Authors:** Aghiles Hamroun, Nihad Boukrout, Christelle Cauffiez, Sandy Fellah, Cynthia Van der Hauwaert, Nicolas Pottier, Romuald Mentaverri, Jeremy Zaworski, Viviane Gnemmi, Jean-Baptiste Gibier, Emmanuel Letavernier, Alexandre Louvet, François Provôt, Rémi Lenain, Mehdi Maanaoui, François Glowacki, Arnaud Lionet

**Affiliations:** Nephrology, Public Health-Epidemiology, Lille University Hospital Center, Lille, France; UMR1167 RID-AGE, Institut Pasteur de Lille, Inserm, Lille University, Lille University Hospital Center, Lille, France; Univ. Lille, CNRS, Inserm, CHU Lille, Institut Pasteur de Lille, UMR9020-U1277 – CANTHER – Cancer Heterogeneity, Plasticity and Resistance to Therapies, Lille, France; Univ. Lille, CNRS, Inserm, CHU Lille, Institut Pasteur de Lille, UMR9020-U1277 – CANTHER – Cancer Heterogeneity, Plasticity and Resistance to Therapies, Lille, France; Univ. Lille, CNRS, Inserm, CHU Lille, Institut Pasteur de Lille, UMR9020-U1277 – CANTHER – Cancer Heterogeneity, Plasticity and Resistance to Therapies, Lille, France; Univ. Lille, CNRS, Inserm, CHU Lille, Institut Pasteur de Lille, UMR9020-U1277 – CANTHER – Cancer Heterogeneity, Plasticity and Resistance to Therapies, Lille, France; Univ. Lille, CNRS, Inserm, CHU Lille, Institut Pasteur de Lille, UMR9020-U1277 – CANTHER – Cancer Heterogeneity, Plasticity and Resistance to Therapies, Lille, France; Amiens University Hospital, Human Biology Center, Amiens, France; UR 7517 UPJV, Pathophysiological Mechanisms and Consequences of Cardiovascular Calcifications (MP3CV), Picardie Jules Verne University, Amiens, France; Inserm, UMR S 1155, Physiology Unit, Hôpital Tenon, Sorbonne Université, Paris, France; Service d'Anatomie Pathologique, Centre de Biologie Pathologique, CHU Lille, Lille, France; Service d'Anatomie Pathologique, Centre de Biologie Pathologique, CHU Lille, Lille, France; Inserm, UMR S 1155, Physiology Unit, Hôpital Tenon, Sorbonne Université, Paris, France; Liver Unit, Lille University Hospital Center, Lille, France; Nephrology, Lille University Hospital Center, Lille, France; Nephrology, Lille University Hospital Center, Lille, France; Nephrology, Lille University Hospital Center, Lille, France; Univ. Lille, CNRS, Inserm, CHU Lille, Institut Pasteur de Lille, UMR9020-U1277 – CANTHER – Cancer Heterogeneity, Plasticity and Resistance to Therapies, Lille, France; Nephrology, Lille University Hospital Center, Lille, France; Univ. Lille, CNRS, Inserm, CHU Lille, Institut Pasteur de Lille, UMR9020-U1277 – CANTHER – Cancer Heterogeneity, Plasticity and Resistance to Therapies, Lille, France; Nephrology, Lille University Hospital Center, Lille, France

**Keywords:** acute hepatitis, FGF23, hypophosphatemia, phosphate diabetes, translational study

## Abstract

**Background:**

Although hepatic production of FGF23 has been suggested in chronic settings, there are no data indicating hypophosphatemia resulting from acute hepatic FGF23 production. Based on two clinical observations of profound hypophosphatemia in the setting of acute hepatitis, our study investigates the hypothesis of acute FGF23 liver expression.

**Methods:**

Retrospective analyses were conducted to estimate FGF23 liver expression both qualitatively (*in situ* hybridization) and quantitatively (relative FGF23 gene expression and protein production) on histological specimens of human and murine acute hepatitis livers, compared with controls of hepatic fibrosis or healthy liver.

**Results:**

The index clinical case involves acute alcoholic hepatitis complicated by profound hypophosphatemia due to phosphate diabetes, revealing a major production of both FGF23 C-terminal fraction (cFGF23) and bio-intact form (iFGF23, 39 751 RU/mL, N: 21–91; and 228.6 pg/mL, N: 22.7–93.1, respectively). A second case of acute hepatitis related to erythrocytic protoporphyria also exhibited comparable abnormalities. In both cases, no other cause of renal phosphate wasting was identified, and the hydroelectrolytic disorders disappeared in parallel with normalization of the liver balance and FGF23 levels. Histological data of acute hepatitis compared with cirrhosis and healthy liver confirmed our hypothesis of hepatic FGF23 overproduction. Furthermore, mouse models showed a significant increase in FGF23 mRNA relative liver expression in acute hepatitis and a moderate increase in cirrhosis, compared with healthy liver (respectively 60.55 ± 16.75 and 3.70 ± 0.87 vs 1.00 ± 0.65, both *P* < .05). These findings were also confirmed at the protein level.

**Conclusion:**

This translational study raises the hypothesis of renal phosphate wasting induced by excessive hepatic production of FGF23 in case of acute hepatitis.

## Graphical Abstract

**Graphical Abstract:**
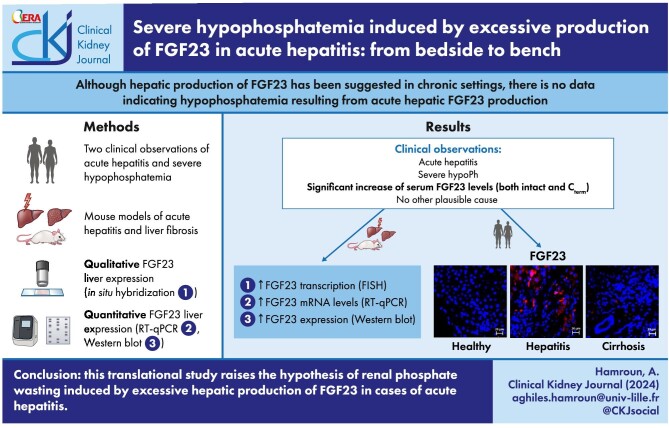

KEY LEARNING POINTS **What was known:**

1. The etiological assessment of hypophosphatemia is complex, particularly in acute hepatitis where several mechanisms may be involved.
2. A few observations of moderate hepatic FGF23 production have been reported in chronic contexts, such as cirrhosis or polycystic fibrosis.

**This study adds:**

1. This translational study reports on two clinical cases of acute hepatitis complicated by profound hypophosphatemia due to renal phosphate wasting, revealing high levels of serum FGF23.
2. We observed a significant increase in hepatic FGF23 expression in human and mouse liver biopsies of acute hepatitis and demonstrated hepatocyte induction of FGF23 mRNA and protein in mouse liver histology specimens of acute hepatitis, compared with cirrhosis and healthy liver.

**Potential impact:**

1. Hypophosphatemia mediated by increased levels of FGF23 is probably underdiagnosed and its screening should be considered in case of unexplained severe hypophosphatemia during acute hepatitis.

## INTRODUCTION

Hypophosphatemia affects approximately 2% of hospitalized patients and may occur in 30% of patients admitted to intensive care units, with a prevalence as high as 80% in case of sepsis [1]. It is associated with a poor prognosis, with an increased risk of cardiovascular events and all-cause mortality [2, 3]. The main recognized mechanisms are decreased intestinal absorption, intracellular redistribution and increased renal excretion. This urinary phosphate excretion is regulated by NaPi2a, NaPi2c and PiT2 cotransporters in the proximal convoluted tubule and by NaPi2b in the thin and thick limbs of the loop of Henle under the influence of Fibroblast Growth Factor 23 (FGF23) [4–6]. FGF23 is a hormone mainly produced by osteocytes and lining cells in bones, and its metabolism depends essentially on phosphate exposure, parathyroid hormone (PTH) and vitamin D system [4, 7]. The biological effect of FGF23 requires both the binding to FGF receptor and to its coreceptor, alpha klotho [6]. FGF23 production is physiologically increased in chronic kidney disease and hemodialysis patients in response to hyperphosphatemia [8]. In contrast, uncontrolled FGF23 production by mesenchymal tumor can induce major renal phosphate waste with hypophosphatemia, responsible for oncogenic osteomalacia [9, 10]. More recently, some data also suggested a production of FGF23 by the liver [11, 12]. An elegant illustration of this hypothesis was presented in 2016 by Wasserman *et al.* through the observation of two infants with end-stage liver disease due to biliary atresia (BA) who developed hypophosphatemia with renal phosphate wasting [11]. The authors observed very high levels of circulating FGF23, all of the abnormalities having been corrected following liver transplantation. Immunohistochemistry thus revealed ectopic overexpression of FGF23 by hepatocytes in the BA liver. Other observations of moderate hepatic FGF23 production have been reported in other chronic contexts, such as cirrhosis or polycystic fibrosis [13, 14].

However, to our knowledge, there is no evidence to date of hypophosphatemia in association with renal wasting secondary to excessive hepatic production of FGF23 in an acute setting. We report here two clinical cases with profound hypophosphatemia revealing major hepatic FGF23 overexpression in the context of acute hepatitis, as confirmed by serum biochemistry and liver biopsy as well as experimental murine models.

## MATERIALS AND METHODS

### Clinical cases

We report two cases of profound hypophosphatemia discovered in the context of acute hepatitis. We detail data concerning age, sex, cause of acute hepatitis, detailed etiological work-up, supplementation therapies and the temporal trajectory of parameters of interest up to 6 months (phosphate imbalance, FGF23 serum level and liver biology). Both patients gave their informed consent for reporting their case and the study was approved by the Institutional Review Board. These observations are reported according to the CARE recommendations.

### Mouse models

Based on these clinical observations, retrospective experimental analyses were conducted on human livers and murine models of acute and chronic hepatitis. All animal care and experimental protocols were approved by the Institutional Animal Care and Use Committee (IACUC) of Lille University (protocol number: 2017110612102233) and are reported according to the ARRIVE guidelines [15]. Manipulators carried out all experimental protocols under strict guidelines to ensure careful and consistent handling of the mice. Animal procedures were performed in C57Bl6/J or Balb/C mice (Janvier Labs). A total of 24 mice were used in this study. Sample size was chosen empirically based on our previous experiences in the calculation of experimental variability; no statistical method was used to predetermine sample size and the number of samples. After 1 week of acclimatization, mice were randomly separated and allocated to control or treatment groups. Cotton sticks were placed in the cages to reduce mouse stress during the entire procedure. As no limit point (drastic weight loss) was reached during the study, no sample mice or data points were excluded from the reported analyses.

#### Bile duct ligation model

Anaesthetized mice (10-week-old male C57BL/6 mice) underwent bile duct ligation (BDL) operation (*n* = 5), which consisted of double ligation with 4–0 silk ligatures and transection of the bile duct, as previously described [16, 17]. The control animal group (*n* = 3) underwent a sham operation, where the common bile duct was exposed but not ligated. Mice were sacrificed 6 days after surgery and livers were collected.

#### CCl_4_-induced liver fibrosis model

The experimental procedure was adapted from previous reports [16, 17]. Briefly, 8-week-old male Balb/C mice (*n* = 10) were injected CCl_4_ (4 mL/kg) dissolved at 5% in corn oil intraperitoneally twice a week while control mice (*n* = 6) were injected vehicle (corn oil). After 6 weeks of treatment, mice were sacrificed and livers were collected.

### Histopathology

Livers were fixed overnight with neutral buffered formalin and then embedded in paraffin. Five-micrometer-thick sections were mounted and stained with Sirius red to assess the degree of fibrosis. These were also stained with Hematoxylin-Eosin-Safran (HES) for optical analysis. Next, sequential tissue sections were labelled with marked anti-CD68 antibody (specific for macrophages and therefore Kupffer cells) for immunofluorescence analysis.

#### RNA-FISH (RNAscope^®^)

Five-micrometer paraffin-embedded liver tissue sections (patients or mice) were first deparaffinized and rehydrated. Fluorescence *in situ* hybridization (FISH) assays were performed by using Multiplex Fluorescent Reagent kit V2 (Advanced Cell Diagnostics) and Opal™ dyes 520, 570 and 690 (Perkin Elmer) as fluorophores according to manufacturer's recommendations. RNAscope probes directed against hsa-FGF23, hsa-NR1H4 (corresponding to FXR), hsa-FGF19, mmu-Fgf23 and mmu-nr1h4 (corresponding to fxr) were obtained from Advanced Cell Diagnostics. The choice of FGF19 is justified by its structural proximity to the FGF subfamily of soluble endocrine proteins and its hepatic expression in the context of acute hepatitis [18]. FXR was used as a positive control since its expression is maintained in healthy liver and decreased in cirrhosis [19]. A DAPI solution (Advanced Cell Diagnostics) was used to stain the nucleus. Acquisition was performed using a microscope slide scanner Axioscan Z1 (Zeiss) at ×20 magnification.

#### Gene expression

Total RNA from mouse liver tissues were prepared as previously described [20]. Reverse transcription was performed on 1 µg RNA using High Capacity cDNA Reverse Transcription kit (ThermoFisher) according to the manufacturer's recommendations. Real-time PCR was performed with Taqman™ Universal Master Mix II, no UNG (ThermoFisher) using the StepOne Plus Real Time PCR System (ThermoFisher). Expression levels of FGF23 gene (assay ID Mm00445621_m1), interleukin (IL)-6 (assay ID Mm00446190), IL-1B (assay ID Mm00434228) and tumor necrosis factor (TNF) (assay ID Mm00434228) were evaluated using the comparative Ct method (2^−∆∆Ct^). Transcript level of PPIA (Mm02342430_m1) was used for normalization as an endogenous control.

### Western blot

Total protein (10 μg) was heated for 10 min at 70°C and loaded onto NuPAGE Novex gels (Thermo Fisher Scientific). After transferring the proteins onto nitrocellulose membranes, the membranes were blocked with 5% milk in TBS-Tween and incubated with anti-FGF23 antibody (1:250; no. 21–6310, Quidel). Visualization of proteins was achieved using horseradish peroxidase–coupled secondary antibodies (1:10 000; Santa Cruz). Signal detection was performed using the ECL Select Chemiluminescence Kit and FUSION FX SPECTRA (VILBER). Membranes were probed with anti-HSP90 antibody (no. 13 119, Santa Cruz) as a normalizer. Data were analyzed with ImageJ.

### Statistics

Data are presented as the mean ±  standard error of the mean (SEM). Differences between groups were assessed using a parametric Student's test (the variances being considered as equal) and using GraphPad Prism 8^®^. (GraphPad Software). Differences were considered statistically significant at a *P*-value of <.05.

## RESULTS

### Case description

**Figure 1:**
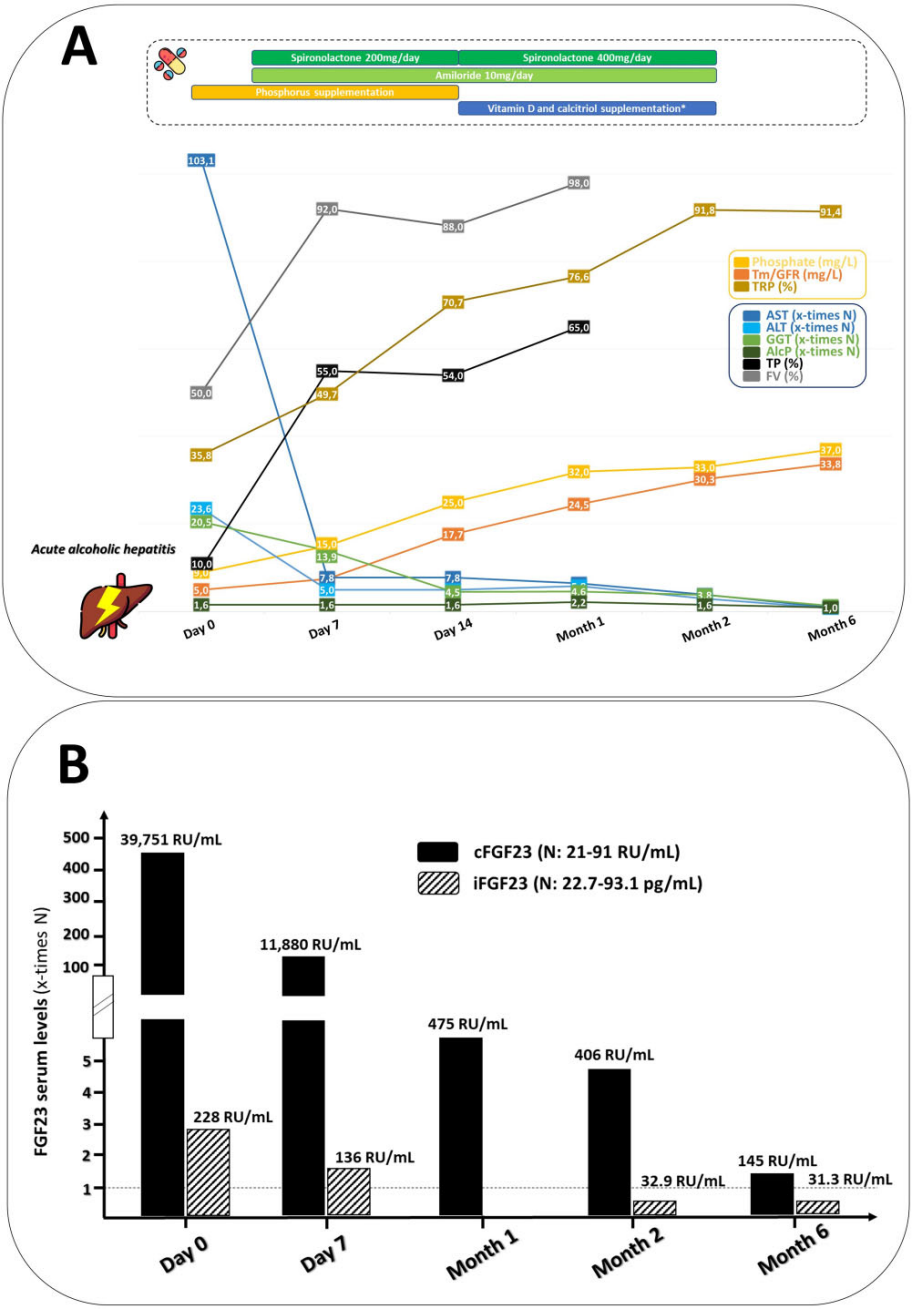
Evolution of liver biochemical parameters and phosphate balance in a patient with acute alcoholic hepatitis (Patient #1). ALT: alanine transaminase; AST: aspartate transaminase; ALP: alkaline phosphatase; GGT: gamma-glutamyl transferase; TRP: total reabsorption of phosphate. *Active vitamin D3 (1 µg of alfacidol per day) + 50 µg of 25OH-cholecalciferol per day.

The index case was a 28-year-old man hospitalized in an intensive care unit due to acute alcoholic hepatitis confirmed by histology. His medical history was marked by hypertension, grade 1 obesity and chronic alcohol abuse. Upon admission to the hospital, the patient had significant abnormalities in liver function, hepatocellular insufficiency and marked bicytopenia (Table 1). On the fifth day of hospitalization, the liver unit team requested nephrologists’ assistance in assessing complex hydroelectrolytic disorders. The patient displayed profound hypophosphatemia and hypokalemia, both of which were challenging to manage despite significant exogenous supplementation (24 g/day of phosphate and 16 g/day of potassium salts in continuous intravenous administration). Overall, the patient displayed signs of complete proximal tubular dysfunction, including phosphate diabetes, hypouricemia, hypokalemia and normoglycemic glycosuria. Renal phosphate wasting was confirmed by a phosphate TmP/DFG level of 5 mg/L upon admission, which increased to 7.5 mg/L on D5. Additionally, the tubular reabsorption of phosphate (TRP) was estimated at 35% (Table 1). Comprehensive investigations ruled out autoimmune diseases, monoclonal gammopathy, overload diseases (e.g. hemochromatosis or Wilson's disease), heavy metal intoxication or any toxic abuse as potential causes. The etiological work-up ultimately revealed a significant increase in circulating levels of both FGF23 C-terminal fraction (cFGF23) and the bio-intact form (iFGF23, 39 751 RU/mL, N: 21–91; and 228.6 pg/mL, N: 22.7–93.1, respectively). Since these measurements were taken at baseline (Day 0), they could not have been affected by the initiation of phosphate supplementation. Complementary PET scanner imaging did not reveal any evidence of a mesenchymal tumor. The clinical course provided evidence supporting the hypothesis of Fanconi syndrome predominating on renal phosphate leak as a consequence of FGF23 excessive secretion in the setting of acute hepatitis, with complete resolution of laboratory abnormalities coinciding with hepatic recovery (Fig. 1).

**Table 1:** Biochemical parameters and complete etiological work-up of the complex hydroelectrolytic disorders presented by Patient #1 (acute alcoholic hepatitis).

Biochemical parameter	Value	Norm
Standard liver work-up		
Hemoglobin	6.8	13–17 g/dL
Platelet count	33	150–400 10^9^/L
White blood cells count	2.69	4–10 10^9^/L
Aspartate transaminase	3093	10–50 UI/L
Alanine transaminase	825	10–50 UI/L
Gamma-GT	924	10–50 UI/L
Alkaline phosphatase	144	40–129 UI/L
Bilirubin	278	1–12 mg/L
TP	<10	>70%
Factor V	51	60–120%
Serum parameters		
Phosphorus	9	25–45 mg/L
Calcium	82	85–105 mg/l
Potassium	2.7	3.5–5.0 mmol/L
Creatinine	8	6–10 mg/L
Uric acid	19	30–70 mg/L
Glucose	0.67	0.65–1.0 g/L
CRP	38	< 6 mg/L
PTH	112	12–88 pg/mL
25(OH) vitamin D	4	>20 ng/mL
1,25(OH) vitamin D	93	15–90 pg/mL
cFGF23	39 751	21.6–91.0 RU/mL
iFGF23	228.6	22.7–93.1 pg/mL
Urinary parameters		
Phosphate TmP/DFG	7.52	25–45 mg/L
Total reabsorption of phosphorus	35.83	>86%
Fractional excretion of phosphorus	64	<20%
Fractional excretion of uric acid	36.5	8–12%
24-h protein	2.62	–
Glucose	1.22	–
Beta2-microglobulin	43.46	<0.202 mg/L
Specific etiological work-up		
Serum copper	1182	600–1700 µg/L
Serum ceruloplasmin	0.52	0.2–0.6 g/L
Serum selenium	59	73–110 µg/L
Serum zinc	0.74	0.5–0.8 mg/L
Serum lead	Undetectable	
Serum cadmium	Undetectable	
Immunological markers		
Antimitochondrial antibodies	Negative	
Anti-LKM1 antibodies	Negative	
Anti-smooth muscle antibodies	Negative	
Antinuclear antibodies	Negative	
Plasma immunoelectrophoresis	Normal	
Kappa/Lambda ratio	0.84	0.26–1.65
Urine copper	181	<20 µg/L
Urine copper/creatinine ratio	169	<12 µg/g
Urine hippuric acid	Undetectable	
Urine delta-aminolevulinic acid	20.6	0–38 µmol/L
Urine amino acids electrophoresis	Normal	

**Figure 2:**
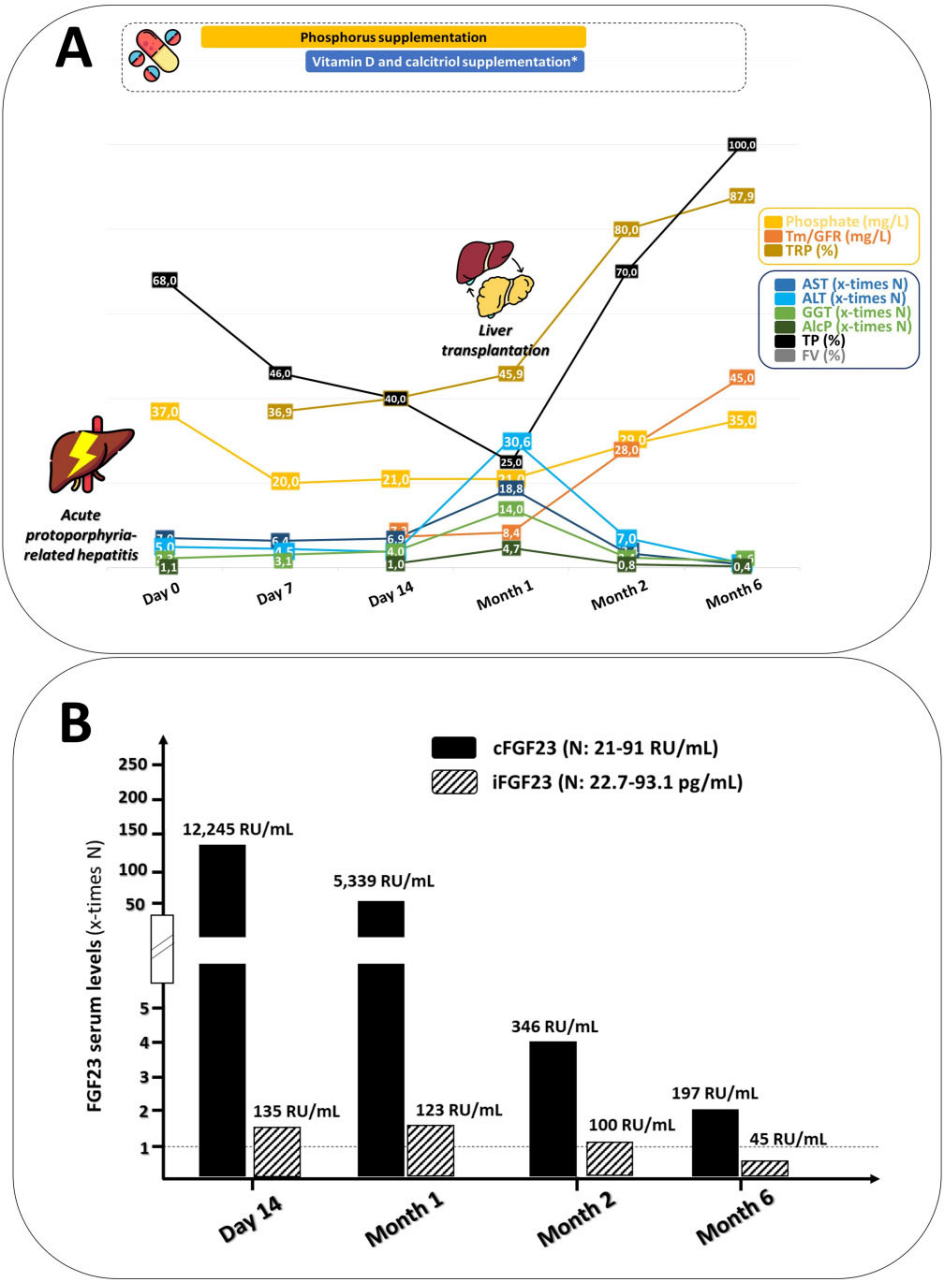
Evolution of liver biochemical parameters and phosphate balance in a patient with acute hepatitis related to erythrocytic protoporphyria, before and after liver transplantation (Patient #2). ALT: alanine transaminase; AST: aspartate transaminase; ALP: alkaline phosphatase; GGT: gamma-glutamyl transferase; TRP: total reabsorption of phosphate. *Active vitamin D3 (1 µg of alfacidol per day) + 50 µg of 25OH-cholecalciferol per day.

A similar evolution was noted in a 66-year-old woman with erythrocyte protoporphyria-related acute hepatitis who received a liver transplant, demonstrating a significant improvement in phosphate balance abnormalities following the transplantation procedure (Fig. 2).

### Human and mouse liver expression of FGF23

**Figure 3:**
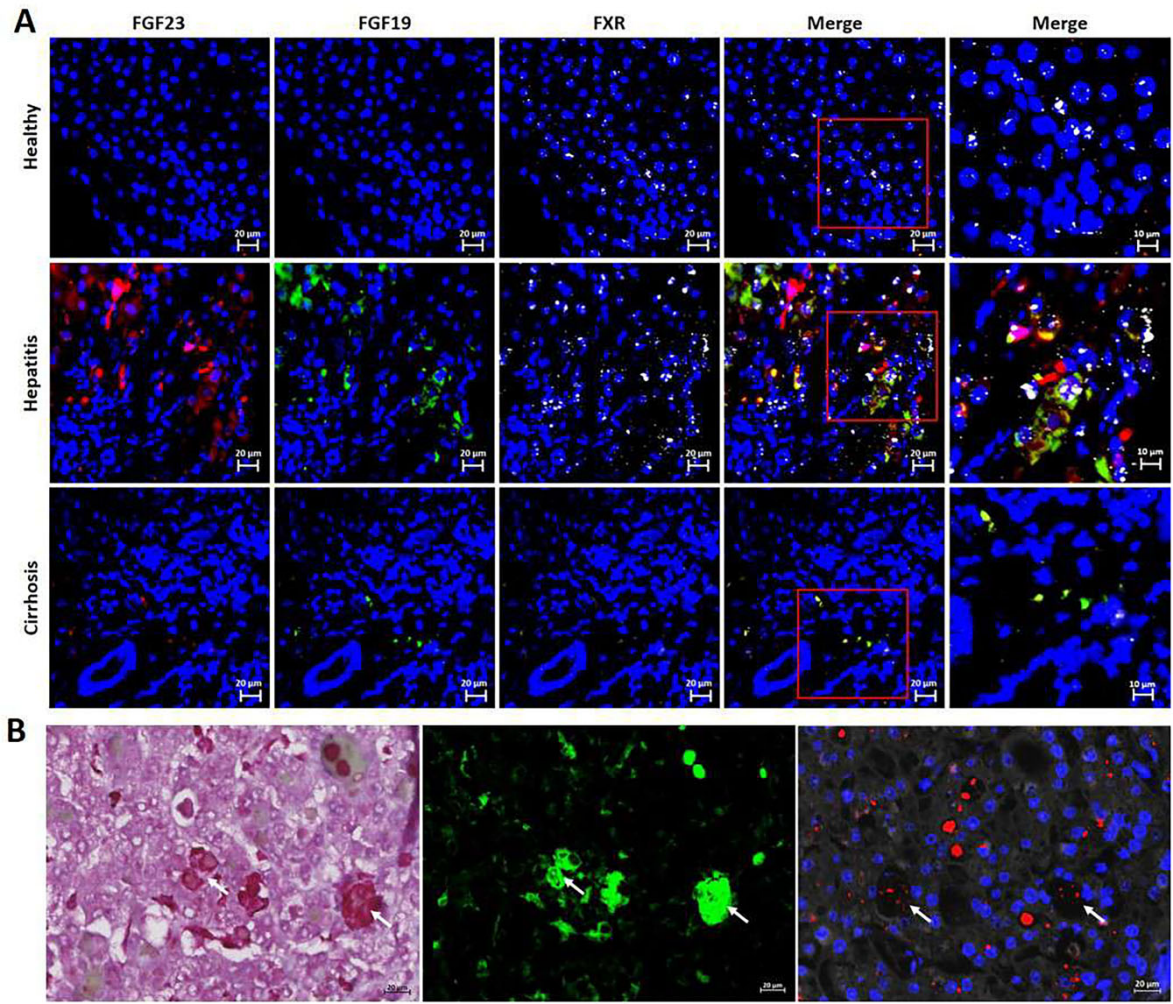
Human liver expression of FGF23 is increased in acute hepatitis. (**A**) RNA–FISH was assessed in human clinical samples. FGF23 (red), FGF19 (green), FXR (white); nuclei were stained with DAPI (blue). (**B**) Representative images of sequential analysis of hepatitis tissue (×400 magnification) with HES staining (left); immunofluorescence after anti-CD68 antibody (middle) and RNA-FISH (right—FGF23 in red, nuclei with DAPI in blue). White arrows indicate Kupffer cells.

In line with the biochemical parameters described in the case reports, we observed that FGF23 mRNA expression was abundant in liver tissue of the patients with acute hepatitis, moderately detected in cirrhotic liver and not detected in healthy liver. FGF23 expression was mainly observed in FGF19-expressing cells. Moreover, as expected, FXR was only expressed in healthy liver and acute hepatitis, but not in cirrhotic liver (Fig. 3A). Immunohistochemical and RNA-FISH analyses of hepatitis tissue revealed that FGF23 mRNA was expressed in both hepatocytes and Kupffer cells (Fig. 3B).

**Figure 4:**
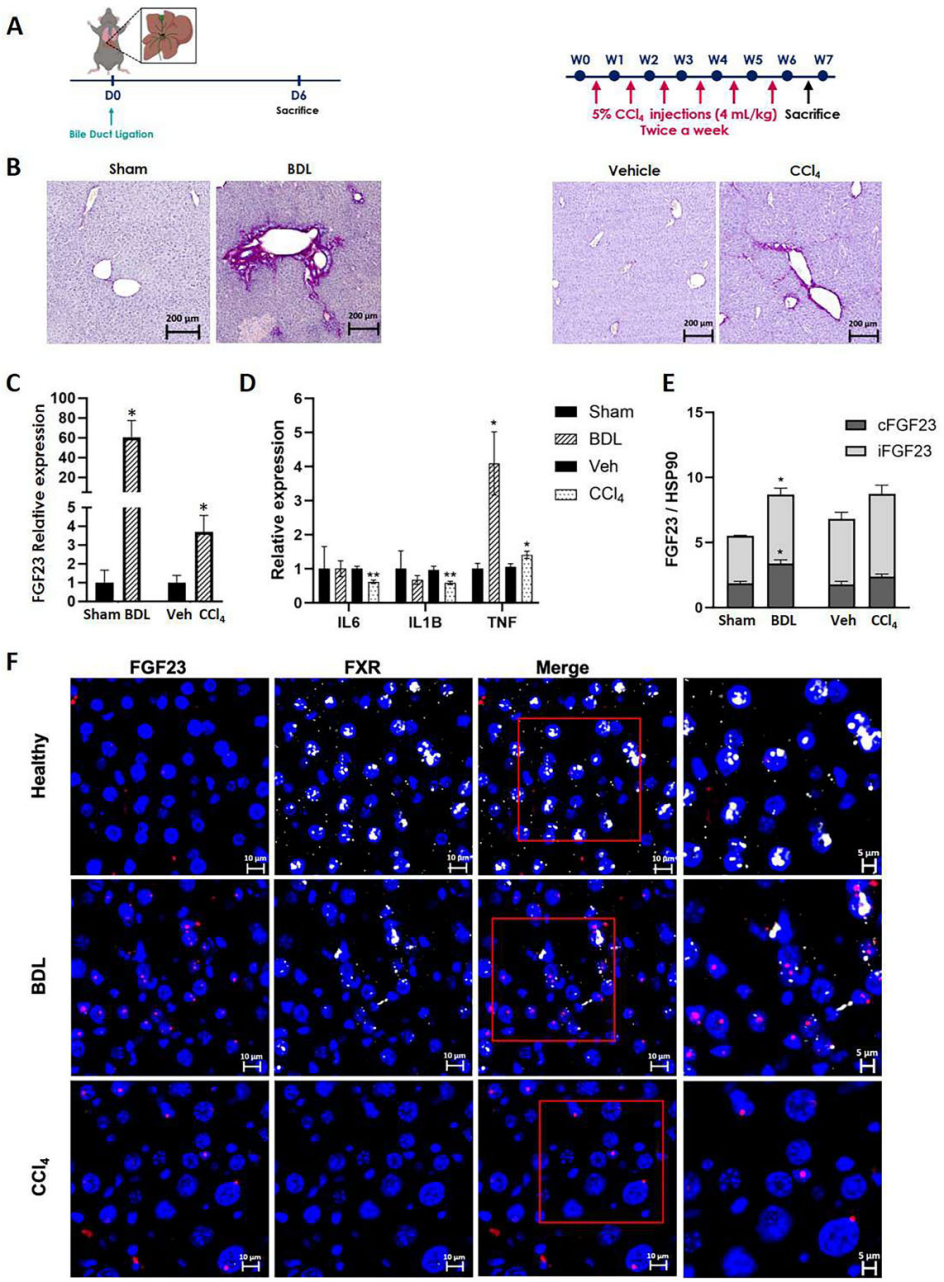
(**A**) Diagram describing the BDL and CCl_4_ administration mouse models. (**B**) Histological assessment of hepatic fibrosis using Sirius red staining. (**C**) Bar charts showing the relative liver expression of FGF23. PPIA was used as a normalizer. (**D**) Bar charts showing the relative liver expression of IL-6, IL-1B and TNF. (**E**) Bar charts showing the quantification of iFGF23 and cFGF23 proteins. HSP90 was used as a normalizer. (**F**) Representative images of RNA–FISH of FGF23 (red) and FXR (white). Nuclei were stained with DAPI (blue). Results indicate the mean ± SEM. *P < .05. Veh: vehicle.

To confirm these data, we retrospectively used two mouse models of liver diseases that induce fibrosis, mimicking acute hepatitis (BDL) and cirrhosis (repeated CCl_4_ administration) (Fig. 4A). Both models produce specific fibrotic lesions (Fig. 4B). BDL triggered a significant upregulation of hepatic FGF23 RNA expression (60.55 ± 16.75 vs 1.00 ± 0.65 in controls, *P* < .05). The CCl_4_ model also showed increased FGF23 expression (3.70 ± 0.87 vs 1.00 ± 0.65 in controls, *P* < .05), but to a lesser extent than BDL (Fig. 4C). We compared the upregulation of FGF23 RNA with several cytokine expressions, finding it proportional to the increase in TNF expression (BDL: 4.09 ± 0.92 vs 1.00 ± 0.15 in controls, *P* < .05; CCl_4_: 1.34 ± 0.10 vs 1.00 ± 0.09 in controls, *P* < .05), but not IL-6 or IL-1 (Fig. 4D). At the protein level, BDL induced overexpression of total FGF23 (8.69 ± 0.76 vs 5.53 ± 0.17 in controls, *P* < .05), including both cFGF23 (3.34 ± 0.28 vs 1.87 ± 0.11 in controls, *P* < .05) and iFGF23 (5.30 ± 0.49 vs 3.65 ± 0.02 in controls, *P* < .05) (Fig. 4E and Supplementary data, Fig. S1). The CCl_4_ model did not show a statistically significant difference. These findings were consistent with RNA *in situ* hybridization, confirming the localization and expression levels of FGF23 RNA in liver tissue (Fig. 4F).

## DISCUSSION

To our knowledge, this study is the first to report on clinical observations of phosphate diabetes in acute hepatitis, as attested by excessive serum FGF23 levels together with hepatic overexpression of FGF23, as compared with findings in normal hepatic tissue or liver cirrhosis. We also observed hepatocyte and Kupffer cells induction of FGF23 mRNA as well as FGF23 protein production in mouse liver histology specimens of acute hepatitis, compared with cirrhosis and healthy liver.

These results are consistent with previous studies that have shown a link between liver disease and hypophosphatemia in an acute setting. For example, a 2016 cohort study of patients undergoing partial hepatectomy found that more than half of them experienced transient hypophosphatemia at Day 2–3 post-surgery [21]. This was more frequent in cases of postoperative hepatocellular failure, and associated with a better prognosis for recovery of liver function. This phenomenon is attributed to elevated parathyroid hormone levels and increased expression of nicotinamide phosphoribosyltransferase in the kidneys. These changes ultimately inhibit sodium-phosphate cotransporters and tubular phosphate reabsorption, already arguing for an acute kidney–liver axis [22]. In another study of 76 patients with acute liver failure, hypophosphatemia was identified in nearly 60% of cases, which was associated with renal phosphate wasting [23]. However, the association between hypophosphatemia, ectopic FGF23 production and liver pathology has been more often documented in the context of chronic liver damage [13, 14]. For example, in a cohort of 200 patients with cirrhosis, Prié *et al.* showed that two-thirds of the cases had increased FGF23 levels, with a mean level of approximately two times normal [13]. These results are in agreement with the FGF23 expression we observed in a mouse model of liver cirrhosis. The presence of FGF23 mRNA in the context of cirrhosis was also observed on human and murine histology specimens in our study. In contrast, we observed a marked increase in FGF23 expression to nearly 60 times normal in acute hepatitis in our mouse model, which also corresponded to the major increases of FGF23 levels observed in our patients in the context of acute hepatitis. Our results suggest that, unlike in chronic kidney disease, acute hepatitis leads to an increase in both iFGF23 production and its cleavage, with the latter being even more pronounced. These findings are consistent with previously described regulatory mechanisms of FGF23 in osteocytes during non-renal inflammation [24, 25]. Preclinical studies have shown that inflammation directly stimulates FGF23 production and simultaneously upregulates its cleavage in osteocytes, leading to FGF23 levels similar to those observed in our study, at 2–3 times the reference for iFGF23 and over 10 times for cFGF23.

Interestingly, we also observed in our cases stigmata of complex tubular dysfunction in the presence of high levels of FGF23. Genetic abnormalities of NaPi2a have been identified as a rare cause of Fanconi syndrome, as reported by Magen *et al.* in 2010 from the observation of two twins [26]. In this publication, SLC34A1 variants were identified as responsible for a NaPi2a loss-of-function, leading to complete proximal tubular dysfunction in these two infants. The underlying mechanism may be intracellular phosphate depletion with consequent insufficient ATP generation and alteration of active tubular transport, which has also been observed in other patients [27, 28] and reported in mice with genetic-related hypophosphatemic rickets [29, 30]. This mechanism is likely underestimated since the presence of phosphate diabetes during acute hepatitis is often not investigated in the presence of other intertwined causes of hypophosphatemia, such as malnutrition, alcohol abuse or intrahepatic transcellular transfer.

From a pathophysiological perspective, the major mechanism proposed to explain hepatic FGF23 production in acute hepatitis is inflammation mediated. It has been demonstrated that hepatocytes produce FGF23 through the activation of estrogen-related receptor gamma (ERRG) by proinflammatory cytokines such as IL-6, IL-1B and TNF-α [31, 32]. Furthermore, other authors have reported that Kupffer cells can also produce FGF23 in response to these same cytokines, as also suggested by our results [33]. The hypothesis of an inflammatory context is supported by our clinical cases and experiments, thus suggesting a potential role of TNF both locally and systemically. This hypothesis is also illustrated in our case by the detection of FGF23 mRNA alongside that of FGF19, the increased expression of which during acute hepatitis is already well established [35]. Additionally, this hypothesis is supported by previous work demonstrating a link between inflammation and increased FGF23 transcription in osteocytes, indicating an already established causative interconnection between inflammation and FGF23 production [24, 25]. Our study thus suggests the potential for a similar phenomenon occurring directly in the liver, the primary site of inflammation during hepatitis.

We acknowledge that this study has limitations due to its reliance on two clinical observations and partial experimental data. Since the experimental data were retrospectively collected from histological specimens, we could not correlate these findings with biochemical data from the mouse models. Specifically, tissue observations could not be matched with blood levels of iFGF23, cFGF23 or phosphate in the mice. However, altogether, our observations already appear to support our hypothesis of a link between FGF23 overproduction and hypophosphatemia, since (i) the complete etiological work-up did not reveal any other plausible causes, (ii) our results were supported by increased serum bio-intact FGF23 levels and the parallel favorable evolution of hepatic and biochemical parameters, and (iii) the liver expression of FGF23 was clearly induced in mouse models of acute hepatitis. Given that vitamin D and phosphate supplementation can influence serum FGF23 levels, we cannot entirely rule out the possibility that the elevated FGF23 levels observed in our patients might be partially reactive to the treatments administered [36]. However, it is important to note that the first measurement for Patient #1 was taken at baseline from samples collected before any treatment. Additionally, these supplements were administered continuously via intravenous infusion, reducing the potential bolus effect on FGF23 levels [37]. Finally, the initial very high FGF23 levels were observed during hypophosphatemia, making it unlikely that the increase was a reaction to phosphate supplementation [37, 38].

As these data only raise a hypothesis, dedicated experimental studies are needed to confirm the link between acute hepatitis, hepatic overproduction of FGF23 and renal-wasting hypophosphatemia. Our results confirm hepatic production of FGF23 but do not clarify its contribution relative to other production sites, such as bones, in an inflammatory context [24, 25]. Therefore, it is possible that acute hepatitis also increases FGF23 production by the osteocytes, which could significantly contribute to the circulating levels measured in our patients and cause their hypophosphatemia. Further complementary studies are also necessary to explore the clinical relevance, frequency and prognostic influence of FGF23-induced hypophosphatemia in acute hepatitis. Although some observations suggest that hypophosphatemia may be associated with a favorable prognosis in terms of liver function recovery, its importance, link with ectopic production of FGF23, and exact role as a biomarker or therapeutic target require further clarification [39].

In summary, this translational study provides possible evidence of hepatic FGF23 excessive secretion in the setting of acute hepatitis contributing to severe hypophosphatemia. These findings may have significant clinical implications and highlight the need for further investigation into the mechanisms underlying FGF23-related hypophosphatemia and its potential role as a therapeutic target for the treatment of acute hepatitis.

## Supplementary Material

sfae307_Supplemental_File

## Data Availability

The data underlying this article will be shared on reasonable request to the corresponding author.
