# Supplementary figures and images for: Severe hypophosphatemia induced by excessive production of FGF23 in acute hepatitis: from bedside to bench
Source: Clin Kidney J. 2024 Oct 9;17(11):sfae307. doi: 10.1093/ckj/sfae307 (PMC11548962; doi:10.1093/ckj/sfae307)

**A**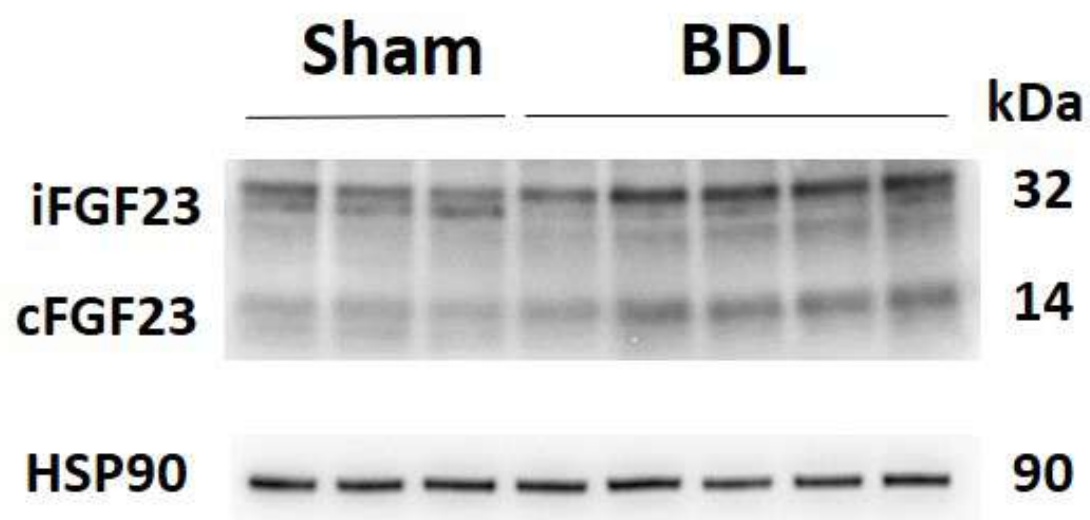**B**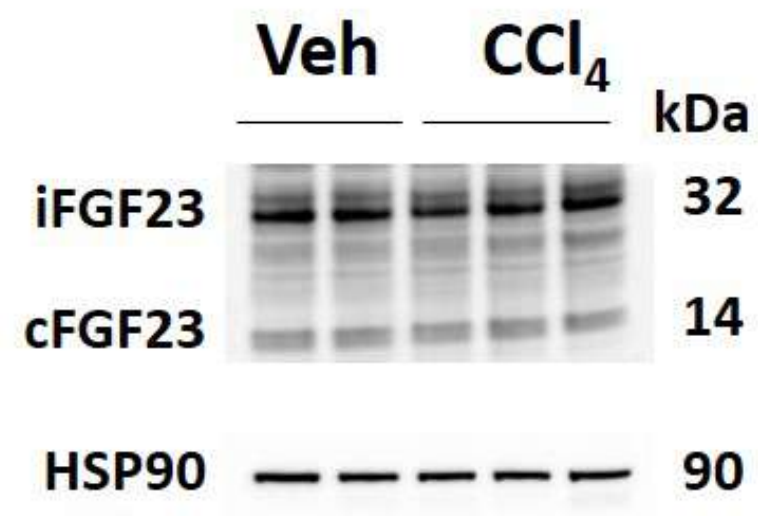

Supplement: sfae307_Supplemental_File [file sfae307_supplemental_file.pdf]
